# The Impact of Neocolonialism on India’s COVID-19 Response

**DOI:** 10.5334/aogh.3587

**Published:** 2022-05-17

**Authors:** Dhruv M. Shah, Mrunmayi Kulkarni, Poonam Mathur

**Affiliations:** 1 University of Maryland School of Medicine, US; 2 Virginia Polytechnic Institute and State University, US

## Abstract

In this paper, we analyze the impact of COVID-19 in India and the nation’s shortcomings in responding appropriately to the pandemic. We discuss how international vaccine inequities, rooted in neocolonialism, and the WHO’s broad recommendation of Global North pandemic responses (i.e., lockdowns, nonspecific social distancing) blunted the effectiveness of India’s COVID-19 response and instead heightened classism, economic turmoil, and unnecessary infection and death from the virus.

*Witnessing the waves of the current pandemic ravage our family members, close friends, and their communities in India was heart wrenching*.

*We gathered the South Asian community at UMB and asked the president of the University of Maryland Medical System Dr. Mohan Suntha a simple question: “What can UMMS do to help?” UMMS had pledged $4.5 million in medical supplies & equipment donations to India & Sri Lanka*.

*We are grateful to UMMS for their contributions. Yet as first-generation citizens of a neocolonial power with higher access to vaccines and healthcare, we still felt a deep cognitive dissonance. The genesis of this viewpoint came from those unresolved feelings*.

## Introduction

SARS-CoV-2 (COVID-19) has been the world’s deadliest, and most globally mismanaged, pandemic in recent history. As of September 2021, it killed a known 4.6 million people and infected 226 million people worldwide [1]. To provide a point of comparison, the world’s last pandemic, the 2009 Swine Flu (H1N1) pandemic, killed an estimated 0.5 million globally [2]. After impacting China, COVID-19 spread extensively in Global North countries including Italy, Spain, and the United States [3]. Given its initial impact on these locations, influential Western media fixated on how the pandemic affected high-income countries (HICs), and many initial control recommendations from organizations such as the United Nations (UN) and its specialized agency, the World Health Organization (WHO), were provided in this context. However, COVID-19 was also ravaging many low- and middle-income countries (LMICs).

India has been particularly devastated by COVID-19. At this time, India has an official death count of about 400,000, but the true death toll has been projected to be an order of magnitude greater: 3 to 3.9 million [4]. For perspective, this estimate is 85% of the today’s reported global death count across all countries [1]. India’s massive underreporting is in part due to the country’s overburdened hospitals [4].

In this paper, we will analyze the impact of COVID-19 in India and the country’s shortcomings in responding appropriately to the pandemic. Paradoxically, although COVID-19 is clearly a global crisis that does not stop at national borders (the word pandemic itself means “all people”), a homogeneous global response is not appropriate or effective. This paper argues that it is imperative to respond to the virus in a country-specific manner to dampen its rampage. Blanket pandemic responses that work in the Global North have not worked in countries such as India given differences in demographics, culture, and economic structure. Further, using India as an example, we argue that international pressure from the WHO and UN to adopt pandemic control and response measures created by and for HICs is a form of neocolonialism. In addition to the non-contextual application of Global North pandemic responses to India, we discuss how international vaccine inequities rooted in neocolonial power dynamics further blunted India’s COVID-19 response, which ultimately heightened classism, economic turmoil, and the unnecessary death of India’s citizens.

## A Summary of India’s COVID-19 Response

On January 30, 2020, the WHO declared the COVID-19 outbreak a “public health emergency of international concern” (PHEIC) [5]. On March 11, 2020, the WHO declared COVID-19 a pandemic, and on March 24, the Government of India announced a 21-day lockdown effective four hours after its announcement [6]. This included the closure of all educational establishments, official mass gatherings, and public transport, as well as physical distancing and a mask mandate in populous public areas [7,8]. India’s infrastructure was not prepared to bear the implications of this sudden announcement. The abrupt lockdown propagated socio-economic disparities, with consequences like food insecurity and unemployment for daily wage workers.

India’s initial lockdown in response to the first wave of infection was extended until June 7, 2020 and was followed by phased relaxations called “unlocks.” After the initial wave, India had seemingly low COVID-19 infection and death rates. India’s Ministry of Health & Family Welfare went from reporting almost 100,000 new COVID-19 cases a day in September 2020 to about 9,100 new cases a day in January 2021 [9]. Experts postulated many theories for how such few cases were occurring in a country with limited social distancing and a population of 1.4 billion, including theories about India’s heat and humidity, increased immunity due to prevalence of other diseases, and India’s young population [9]. Unfortunately, within 3 months, by March 2021, India experienced a second wave of COVID-19 cases and deaths much more profound than the first.

By April 9, 2021, India had the highest number of daily cases globally. This second wave of COVID-19 was, in part, due to double- and triple-mutant strains of SARS-CoV-2 that were more pathogenic than initial strains [9,10]. As a result, there was a dramatically reduced supply of essential treatments and oxygen in India, as well as many other LMICs, resulting in an increased death rate in the younger population [9].

India’s COVID-19 response differed during the second wave compared to the first, due to the shift in responsibility from the federal to state governments. In India, public health measures are typically the duty of the state governments. During the first wave, the federal government asserted its authority to impose a nationwide lockdown. While technically a violation of India’s constitution, this decision arose organically in response to the severity of the COVID-19 crisis. It was rationalized using the precedent set by the Epidemic Disease Act of 1897, as discussed in the next section. However, during the second wave, states exerted their constitutional power and took responsibility for the public health response, which limited their access to key federal resources. As a result, states were unable to scale up key public health interventions, such as vaccination strategies for COVID-19 [11]. This was compounded by inherent public health roadblocks present in India, such as regional variations in health literacy, healthcare inequity, and poor risk perceptions [12]. The state government responses were blunted further by numerous mass gatherings in religious settings, festivals encouraging mass participation, political rallies, and protests, all of which accelerated infection and mortality rates [11,12].

## India’s Colonial History

India’s colonial history is crucial to understanding certain gaps in India’s COVID-19 response. The formal occupation of the British crown in India began in 1858 and lasted until 1947. During this occupation, Indians were banned from holding higher positions of power in the central government. Even local Indian authorities were hand-picked based on their loyalty to the British. India gained its hard-fought independence on August 15, 1947. Thus, the Republic of India is just 75 years old, and still establishing itself economically and politically in today’s world.

India’s relative youth means it is inexperienced with managing infectious outbreaks. The Bombay Plague epidemic of 1896 and the 1918 flu pandemic both occurred during British rule. During the former outbreak, the British passed the Epidemic Disease Act of 1897, which allowed the colonial state and central government to impose dominion over regional Indian authorities [13]. This same Act was adapted and used to authorize federal intervention during India’s first wave response in March 2020. While this released federal funds to fight the pandemic, it also limited the ability of states to craft locally appropriate control measures. Such indiscriminate reuse of a colonial era Act failed to recognize that both federal and state responses were needed to handle COVID-19 in India. In retrospect, the most effective response would have been federal support of state-led local solutions.

## How Classism and Neocolonialism Blunted India’s COVID-19 Response

Internal classism (i.e., discrimination against those in a lower social class) and external neocolonialism blunted the effectiveness of India’s COVID-19 response through two mechanisms: 1) international encouragement for the blanket application of Global North pandemic responses irrespective of India’s unique challenges, and 2) global vaccine inequalities resulting in unnecessary morbidity and mortality for India’s citizens. The former represents an ideological neocolonialism, as the blind application of Global North strategies magnified classism in India. The latter highlights resource-based neocolonialism, as Global North countries secured costly vaccine availability for their own nations with little regard to LMICs like India.

### The Lack of an India-centered Pandemic Response

After the COVID-19 PHEIC was declared in January 2020, the WHO announced, “all countries should be prepared for containment, including active surveillance, early detection, isolation and case management, contact tracing and prevention of onward spread of [SARS-CoV2], and to share full data with WHO” [5]. These containment measures, outlined by the WHO’s International Health Regulations (IHR), included nationwide lockdowns, social distancing, and closing public transport. All these measures were adopted by India, despite the lack of space and resources that made it difficult to implement such measures. Specifically, India’s vast population and cultural norms made universal lockdown and social distancing challenging. For instance, the city of Mumbai has the same population as Australia, however it only has 0.05% of the area [14]. Additionally, 40% of all Indian homes are comprised of a single room as living space [15]. Such high-density urban areas and living conditions with multi-generational households make it difficult to quarantine symptomatic individuals. Research has demonstrated that dense housing capacity and decreased housing size increases the risk for testing positive for COVID-19 by 31% [16]. Limited space also complicated sale of food in urban areas where informal vendors and markets are the norm. Food vending in several parts of India was shut down due to crowding in limited shop spaces creating a hardship for vendors and consumers alike [17,18].

Normative social distancing recommendations appropriate in HICs were equally difficult to achieve in rural Indian settings, where families often share a single water source and worked in close proximity on farms. Strong cultural and religious traditions throughout India also made it difficult to enforce social distancing policies. From March to April 2021, a sequence of “super-spreader” mass congregations were permitted by Prime Minister Narendra Modi, including the Kumbh Mela religious festival, which had an estimated attendance of 9.1 million pilgrims [19]. These gatherings likely precipitated India’s second COVID-19 wave [19].

The WHO and IHR have been criticized for providing guidelines which focus on European and North American HIC’s and overlook the lack of resources in Global South LMICs such as India [20]. IHR’s recommended public health responses are not appropriate for countries with fewer resources nor presented with multiple implementation options for varied settings [21]. For instance, in 2020 India had only one bed per 1,000 patients—significantly fewer than WHO’s prescribed standards of three beds at minimum per 1,000 patients [22]. Despite knowledge of these limitations before the COVID-19 crisis, the WHO did little to modify their recommendations for LMIC’s accordingly.

### Classism during India’s COVID-19 Response

Western-centric pandemic measures specifically affected the Indian labor sector. Over one-third (37%) of India’s population consists of migrant workers who lack job security and financial support from the government [23]. India’s sudden lockdown in March 2020, which shut down factories and public transportation, pushed millions of migrant workers into unemployment [6]. The cessation of public transportation prevented these workers from returning to their villages. Migrant workers were forced to walk anywhere between 10 to 1000 km with minimal food and water. A month into the shut-down, the Indian government operated a limited number of “Shamrik Special” trains to help the migrant workers return home. While Global North countries such as the US could more successfully use social distancing as one primary method to slow the spread of the virus, a large group of Indian citizens had to use crowded public transportation to relocate.

At the end of March 2020, the Indian government announced the equivalent of a $22.5 billion USD stimulus package for vaccine development, loans for businesses, and food security/cash transfers. A component of the package was expansion of India’s National Food Security Act (NFSA) to provide extra monthly allocations of grain to 80 million migrant workers stranded in different states. Unfortunately, in order to redeem this relief, eligible citizens had to provide a ration card that only 59% of covered Indians hold [24]. This gap in ration card coverage is due to NFSA’s use of data from the 2011 census, which did not account for a population growth of over 150 million [24]. The next census, planned for 2021, has been delayed indefinitely due to COVID-19. This meant the NFSA’s COVID-19 effort excluded an estimated 105 million people in need of food security, with no resolution in sight [24].

### Vaccine Neocolonialism

In December 2020, vaccine administration started in multiple HICs with little regard or planning for vaccine availability in LMICs. This approach has been labeled “vaccine colonialism,” or “vaccine apartheid.” The director-general of the WHO Tedros Adhanom Ghebreyesus described vaccine apartheid to the public on May 2021: “HIC’s account for 15 percent of the world’s population, but have 45 percent of the world’s vaccines. LMIC’s countries account for almost half of the world’s population but have received just 17 percent of the world’s vaccines” [25].

Even after securing and distributing vaccines for their countries, powerful Global North countries such as those in the European Union and the US continued to prioritize their own economic and health interests by declining to agree with the Trade Related Aspects of Intellectual Property Rights (TRIPS) waiver, proposed in October 2020 [26]. Supported by the Global South, including India and much of South America, TRIPS is an agreement under the World Trade Organization (WTO) established on January 1, 1995, protecting intellectual property (IP), and in turn resolving trade disputes [27]. The October 2020 proposed TRIPS waiver would have terminated IP protections regarding all aspects of COVID-19 vaccine production. The waiver was sought in order to allow countries to manufacture and distribute any vaccine, as long as they had the resources. However, to date, multiple HIC’s still have not agreed to the waiver, citing “what is needed most urgently is a massive drive of technology transfer, capacity expansion, and supply line coordination to bring vaccine supply in line with global demand” [28].

Shortly after the start of the pandemic, several Global North countries invested in efforts to finance and develop a vaccine, including “Operation Warp Speed”, in the U.S. As vaccines were developed, temporary approvals were given by regulatory agencies, and vaccination campaigns commenced. It is notable that while HICs negotiated with each other to purchase vaccines, there was very little discussion regarding support for LMICs. Critics have noted that this highly nationalistic and protective approach has failed “to allocate vaccines to countries with the greatest need and greatest potential for harm reduction, unnecessarily prolonging the global pandemic and causing many preventable deaths” [29]. As of September 14, 2021, only 13.2% of India’s population had been fully vaccinated, while in the US however 54.5% of the population was fully vaccinated [1].

This “vaccine neocolonialism,” put Global South countries far behind the recovery curve and has arguably backfired on HICs as the virus continues to mutate in locations where vaccines are not available. By June 2021, the COVID-19 death rate globally had topped that of 2020, as countries like India that did not have access to vaccines underwent a second wave [30]. India’s next wave birthed more virulent strands of COVID-19 like the Delta variant, the latest variant at the time of this report, which is spreading rampantly around the world.

To combat inequitable vaccine distribution, the UN launched the COVAX program which made its first international delivery on February 24, 2021 [31]. COVAX was created to boost the world’s odds of effectively creating COVID-19 vaccines and manufacture them in the amounts required to end the COVID-19 pandemic, and guarantee that a country’s ability to pay is not a barrier to accessing them [31]. The COVAX program as created seemed to be an important step to decolonizing global vaccine distribution and achieving global herd immunity. COVAX primarily aims to be financed by HIC’s, who make advance payments to vaccinate 10–50% of their high priority population as vaccines become available. LMIC’s are in turn funded by the COVAX Advance Market Commitment (AMC) [32]. In theory, all countries would vaccinate at the same global rate under the COVAX plan, regardless of their ability to pay. Despite unequal global vaccination rates, the COVAX program has been successful in giving LMICs access to vaccines much faster than they would have otherwise, reaching 100 nations within 2 months of its original delivery date [31]. In spite of the country’s low vaccination rate, India’s Serum Institute produced a majority of COVAX’s vaccine reserves, but vaccine export halted for over 6 months to concentrate on domestic vaccination during the second wave [33]. This move substantially curtailed COVAX’s ability to deliver on its promised 2021 dose distribution around the world. While such resource nationalism seems to oppose our call for a decolonized vaccine distribution process, we maintain it is justifiable given India’s LMIC status in the global COVID crisis. A nationalistic action by an LMIC versus an HIC should be given more leeway based on the many barriers to an effective COVID response enumerated in this very text, such as ill-advised lockdowns, transportation shutdowns, high population density, and delayed vaccine access compared to HICs. These barriers resulted in deaths exponentially higher than official counts. As such, India needed to reserve as many vaccines as possible for its citizens, conceivably beyond official allocation estimates.

The role of international organizations in pandemic response cannot be overstated. India responded quickly to WHO’s pandemic declarations and recommendations, but those recommendations fell short of their promise given their lack of contextual implementation strategies. The limited, but important, success of the COVAX program further demonstrates the potential power of global collectives to support global health equity, however, refinements and actionable guidelines are necessary to increase and enforce HIC support for these efforts.

## Conclusion

In just a few months, the Delta (B.1.617.1) variant emerging first in India has risen as the most infectious variant of SARS-CoV-2 to date. This variant serves as a cautionary tale—not only is it morally wrong for countries with rich histories of colonial power and generational wealth to overlook the necessity of an equitable global COVID-19 response, but doing so also has demonstrable dangerous consequences to their own populations.

The WHO must support LMICs in developing country-appropriate pandemic responses with more specific and culturally sensitive guidelines. In addition, a decolonial approach is required, which questions existing power structures within the WHO and other international organizations and dismantles lasting colonial influences that still impede former colonies like India from mounting context specific pandemic responses. Individual Global South governments like that of India must reflect on and invest in decolonizing their public health structures as well as federal and state pandemic approaches. We fear that Global North countries will not change their behavior for purely altruistic motives. As such, we call on representative global organizations to create and enforce mechanisms that ensure ethical and equitable pandemic responses programs, particularly in the area of vaccine development and distribution. Additional resources must be allocated to those countries that are working to outgrow the colonial legacies that abated their development while colonial powers flourished. This is the only way to reduce global disease burden and the spread of virus variants.

The UN and the WHO must develop more enforcement mechanisms to ensure compliance with collective programs such as COVAX. COVAX’s model is a positive step for global vaccine equality and reducing COVID-19 spread. However, we endorse the Fair Priority Model as a better and equitable model for allocation of vaccines proportional to the emergent needs of each country [29]. In theory, this model would vaccinate the most vulnerable populations first to prevent premature deaths. Next, it would try to reduce economic and social costs by vaccinating low-income populations to narrow the poverty gap, in turn increasing the country’s gross national income [29]. Lastly, it would focus on ending community spread by vaccinating places that have the most uncontrolled spread [29]. This modification prioritizes the most disadvantaged nations and is not only a more ethical method of vaccine distribution, but also more efficiently reduces the global burden of future pandemics.

The future development and implementation of infectious disease surveillance, detection, and response must come from an in-depth analysis of India’s population, their needs, their strengths, and their limits. The calamity of this pandemic has laid bare internal structures of classism, sociocultural disparities, and the developing nature of India’s economy and infrastructure. Future studies must analyze how the UN, WHO, and HICs can decolonize the global pandemic response framework and support LMICs as full partners with contextual needs and unique strengths. This is the challenge and promise of decolonized global health.
